# Sequential ^18^F-AV45/^18^F-AV1451 dual-tracer brain PET imaging in Alzheimer’s disease: amyloid-tau deposition, diagnostic performance, cognitive associations, and modulation by APOE ε4

**DOI:** 10.3389/fneur.2026.1877217

**Published:** 2026-06-19

**Authors:** Guomei Hu, Lijuan Feng, Limeng He, Nan Liu, Hao Wang

**Affiliations:** 1 Department of Nuclear Medicine, The First People’s Hospital of Liangshan Yi Autonomous Prefecture, Xichang, Sichuan, China; 2 Department of Nuclear Medicine, Sichuan Provincial People’s Hospital, University of Electronic Science and Technology of China, Chengdu, Sichuan, China

**Keywords:** Alzheimer’s disease, amyloid-tau deposition, APOE ε4, cognitive function, dual-tracer PET

## Abstract

**Objective:**

Alzheimer’s disease (AD) is neuropathologically defined by amyloid-*β* (Aβ) plaques and tau neurofibrillary tangles. The AT(N) framework underscores the importance of *in vivo* dual-pathology imaging, yet large-scale clinical data on sequential ^18^F-AV45 (Aβ) and ^18^F-AV1451 (tau) PET remain limited. This study aimed to characterize Aβ–tau deposition, evaluate the pathological discriminatory performance of dual-tracer PET for typical amnestic AD, investigate cognitive correlations, and examine the modulatory role of APOE ε4 in a large clinical cohort.

**Methods:**

We conducted a retrospective cross-sectional study of 438 participants, including 325 AD patients, 68 mild cognitive impairment (MCI) patients, and 45 healthy controls (HC). All underwent sequential ^18^F-AV45 and ^18^F-AV1451 PET/CT. SUVRs were calculated in whole brain and 10 predefined regional ROIs, with the inferior cerebellar cortex as reference. Z-score normalization and binary logistic regression models were adopted to combine dual-tracer SUVR data for diagnostic performance assessment. Cognitive assessments included MMSE, MoCA, and ADAS-Cog. Statistical analyses comprised ANOVA, ROC (DeLong’s test), Pearson correlation, and multivariable linear regression.

**Results:**

Whole-brain and regional SUVRs for both tracers were significantly higher in AD than in MCI and HC (all *p* < 0.001), with peak uptake in the temporoparietal lobe. The dual-tracer strategy with standardized combination methods yielded superior pathological discriminatory efficacy for typical amnestic AD compared with single tracers, with an AUC of 0.97 for AD versus HC and 0.93 for AD versus MCI. Tau deposition showed stronger cognitive correlations than Aβ. Tau burden and APOE ε4 were independent predictors of cognitive impairment. Aβ and tau SUVRs were strongly correlated (*r* = 0.65–0.81), most prominently in the precuneus and inferior temporal gyrus. APOE ε4 carriers exhibited significantly higher Aβ and tau deposition.

**Conclusion:**

Sequential ^18^F-AV45/^18^F-AV1451 dual-tracer PET enables accurate *in vivo* characterization of AD-related Aβ–tau pathology, and standardized combined analysis further improves its ability to distinguish typical sporadic amnestic AD from other groups. Tau is an independent driver of cognitive decline, while Aβ remains relevant for amyloid-targeted therapies. APOE ε4 modulates Aβ–tau interactions. Dual-tracer PET is a robust tool for clinical AD evaluation, pathological staging, and therapeutic monitoring of typical amnestic AD.

## 1 Introduction

Alzheimer’s disease (AD) represents the most prevalent neurodegenerative disorder and the leading cause of dementia worldwide, marked by progressive cognitive deterioration and two defining neuropathological features: extracellular deposition of *β*-amyloid (Aβ) plaques and intracellular accumulation of hyperphosphorylated tau protein forming neurofibrillary tangles (NFTs) (1, 2). The AT(N) framework is a widely accepted biological classification system for Alzheimer’s disease that integrates three core biomarker categories: A (amyloid-*β* pathology), T (tau pathology), and N (neurodegeneration or neuronal injury). The AT(N) biological framework proposed by the National Institute on Aging–Alzheimer’s Association (NIA–AA) has become the foundational standard for the biological definition and pathological staging of AD (3), and molecular positron emission tomography (PET) imaging has emerged as an indispensable *in vivo* tool for the quantitative detection of both Aβ and tau pathology (4).

^18^F-AV45 (florbetapir) is a well-validated Aβ-specific PET tracer (5, 6) widely used in clinical and research settings. ^18^F-AV1451 (flortaucipir) is an established tau-specific tracer whose imaging findings closely parallel Braak staging of NFT pathology (7, 8). It is well recognized that amyloid PET provides a sensitive marker of early AD biology (A+), including in cognitively unimpaired individuals at the preclinical stage (3, 9). However, amyloid-positive status alone does not predict the degree of cognitive impairment, as Aβ accumulation may precede symptom onset by decades and can be detected in a proportion of cognitively normal elderly individuals (10, 11). In contrast, tau pathology more closely mirrors symptom severity and is considered the proximate driver of neurodegeneration and cognitive decline (12, 13).

Within the AT(N) framework, sequential use of amyloid PET followed by tau PET for biological staging is well established in research cohorts (14, 15). Nevertheless, several important gaps remain. First, most combined amyloid-tau PET studies were performed in highly selected research or trial cohorts with small sample sizes (generally <150 participants), limiting their generalizability to routine clinical practice (9, 14, 16, 17). Earlier work mostly adopted single-tracer PET or focused on specific patient subgroups, rather than unselected consecutive clinical cohorts. Additionally, few large-scale studies have assessed the incremental diagnostic value of sequential ^18^F-AV45 and ^18^F-AV1451 dual-tracer PET in real-world clinical settings. Current literature also lacks population-based, real-world evidence on the diagnostic performance, regional pathological patterns and cognitive correlations of this dual-tracer protocol (10, 18, 19). Second, although Aβ burden quantified by ^18^F-AV45 SUVR has been linked to cognitive function across the AD–MCI–HC continuum, relevant studies were mostly based on small, highly selected cohorts or single-tracer designs without concurrent tau measurement (20). Large real-world studies using sequential ^18^F-AV45/^18^F-AV1451 dual-tracer imaging to differentiate and compare Aβ-cognition and tau-cognition associations remain scarce. Third, multiple studies have confirmed that APOE ε4 modulates Aβ deposition and tau accumulation independently (21–25), yet most relied on single-tracer PET, small/selected cohorts or analysis of a single biomarker. Few large real-world clinical studies have adopted sequential ^18^F-AV45/^18^F-AV1451 dual-tracer PET to systematically explore how APOE ε4 affects Aβ and tau SUVR profiles and their interactions simultaneously (26). The combined effects of APOE ε4 on dual-tracer signatures in large clinical cohorts therefore remain poorly defined and warrant further investigation.

The specific gap that this study addresses is therefore threefold: (i) to validate the additive diagnostic value of sequentially acquired ^18^F-AV45 and ^18^F-AV1451 PET in a large single-center cohort; (ii) to characterize the independent associations of Aβ and tau burden with cognitive performance; and (iii) to examine the Aβ–tau interaction and the modulatory influence of APOEε4 on both tracers’ deposition patterns.

## 2 Materials and methods

### 2.1 Study participants

A total of 438 subjects who received clinical evaluation at our hospital between February 2022 and December 2025 were retrospectively enrolled, including 325 patients with AD, 68 patients with MCI, and 45 HC individuals. All participant enrollments and clinical diagnoses were completed independently prior to PET imaging, without referencing any PET biomarker results. Diagnoses of AD and MCI were established in accordance with the 2021 NIA–AA diagnostic criteria (27) and the 2018 Chinese Guidelines for the Diagnosis and Treatment of Dementia and Cognitive Impairment (28). Differing from the 2024 NIA-AA biological AD definition that takes amyloid biomarkers as core diagnostic indicators, our clinical enrollment relied on phenotypic symptoms and neuropsychological manifestations. All AD patients demonstrated positive visual and quantitative ^18^F-AV45 uptake and exhibited a typical amnestic phenotype, defined clinically by prominent and disproportionate impairment of episodic memory function on standardized neuropsychological tests relative to other cognitive domains, consistent with the classic phenotype of sporadic amnestic AD. Patients with atypical variants such as posterior cortical atrophy, frontal variant AD, or AD with parkinsonism were excluded. Notably, this amyloid-positive enrollment criterion may introduce circularity when evaluating standalone ^18^F-AV45 diagnostic performance, as the same tracer was used to define the study cohort. HC subjects showed no evidence of cognitive impairment and had no family history of cognitive disorders.

Inclusion criteria consisted of age between 50 and 85 years, completion of both ^18^F-AV45 and ^18^F-AV1451 PET/CT imaging, neuropsychological assessment performed within 1 week before imaging, and availability of APOE ε4 genotyping results. Exclusion criteria included concurrent neurological or psychiatric disorders, severe systemic medical illness, structural intracranial lesions, and a history of traumatic brain injury or cranial surgery. APOE ε4 status was classified as carrier for participants with at least one ε4 allele and non-carrier for those with no ε4 alleles. The study protocol was approved by the Medical Ethics Committee of our hospital (Approval No. 13, January 20, 2022). Written informed consent was waived due to the retrospective and de-identified nature of the study design.

### 2.2 Neuropsychological assessment

All participants underwent comprehensive neuropsychological testing administered by trained and certified psychologists within 1 week before PET imaging. The assessment battery included the Mini-Mental State Examination (MMSE), Montreal Cognitive Assessment (MoCA) and Alzheimer’s Disease Assessment Scale-Cognitive Subscale (ADAS-Cog). Final cognitive scores were calculated as the mean of two consecutive evaluations to minimize measurement variability and enhance reliability.

### 2.3 PET/CT image acquisition

^18^F-AV45 and ^18^F-AV1451 were synthesized under Good Manufacturing Practice (GMP) conditions with radiochemical purity exceeding 95%. All imaging was performed using a Siemens Biograph mCT Flow 64 PET/CT scanner. Participants fasted for a minimum of 6 h before receiving an intravenous injection of 185.0 ± 18.5 MBq of either tracer. PET acquisition commenced at 60 min post-injection for ^18^F-AV45 and at 90 min for ^18^F-AV1451, consistent with established optimal imaging windows. The two PET scans were separated by an interval of 1–2 weeks to minimize cross-tracer interference. CT acquisition parameters included 120 kV, 150 mAs, and 2 mm slice thickness. PET data were acquired over 15 min and reconstructed using TrueX+time-of-flight (TOF) algorithms with 5 iterations, 21 subsets, and 2 mm slice thickness.

### 2.4 Image processing and quantitative analysis

PET images were processed using a Siemens MMWP TrueD workstation. No structural MRI was available for individual participants; therefore, PET images were directly spatially normalized to the Montreal Neurological Institute (MNI152) standard brain template using rigid body transformation without non-linear warping. Partial volume correction (PVC) was not applied, consistent with most clinical routine and ADNI-aligned protocols for dual-tracer SUVR quantification. The lack of PVC may lead to slight underestimation of tracer uptake in small atrophic regions such as the hippocampus and entorhinal cortex, but all groups and regions were analyzed identically to ensure valid comparative statistics. The automated region-of-interest (ROI) atlas supplied with the MMWP TrueD software (based on the Hammersmith atlas and AAL atlas) was used for anatomical labeling and quantitative ROI definition. Gaussian post-reconstruction smoothing (FWHM = 6 mm) was applied to all PET images to improve signal-to-noise ratio and standardize spatial resolution. Registration quality was visually inspected slice-by-slice by two independent nuclear medicine physicians to ensure accurate anatomical alignment between PET and the standard template. The inferior cerebellar cortex, excluding the vermis and adjacent anterior vermis gray matter, was used as the reference region for SUVR calculation for both tracers, in accordance with ADNI protocols. SUVR was defined as the mean standardized uptake value (SUV) of a target regions of interest (ROIs) divided by the mean SUV of the reference region.

Quantitative analysis was performed for the whole brain and 10 individual ROIs, including the frontal, temporal, parietal, occipital, and insular lobes, amygdala, hippocampus, entorhinal cortex, precuneus, and cingulate gyrus. Individual ROIs were selected to enable direct regional comparisons between Aβ and tau deposition and to support clinically interpretable regional quantification.

Visual interpretation was conducted independently by two senior nuclear medicine physicians who reviewed all scans and were blinded to all clinical data. Tracer positivity was defined as abnormal radiotracer uptake in at least three consecutive slices in any brain region. Discrepancies between the two readers were resolved by thorough joint discussion and consensus review; a third reader was not utilized. Optimal SUVR cut-off values were determined using Youden’s index to maximize diagnostic performance. Partial volume correction was not applied in the analysis. Given the modest spatial resolution of clinical PET scanners and potential age-related brain atrophy, small ROIs including the hippocampus and entorhinal cortex may be particularly susceptible to partial volume effects and underestimation of true tracer uptake, which is acknowledged as a methodological limitation.

### 2.5 Dual-tracer combined score

Considering the distinct biological targets, dynamic ranges and binding properties of the two radiotracers, we adopted two statistically standardized combination strategies for comprehensive analysis to ensure robust and unbiased diagnostic evaluation:

(1) Z-score normalization combined score: First, whole-brain SUVR values of ^18^F-AV45 and ^18^F-AV1451 were separately converted to Z-scores using the mean and standard deviation of all enrolled participants, to unify the data distribution and eliminate differences in dynamic ranges between tracers. The Z-score combined score was calculated as the arithmetic mean of the two standardized Z-scores. This method standardizes raw data on the same scale, enabling fair integration of Aβ and tau pathological information.
(2) Binary logistic regression combined model: Taking the diagnostic grouping (AD vs. HC, AD vs. MCI) as the dependent binary variable, whole-brain ^18^F-AV45 SUVR and ^18^F-AV1451 SUVR were incorporated as independent variables to construct binary logistic regression models. The predicted probability output by the model was used as the dual-tracer combined diagnostic index for subsequent ROC analysis. This multivariate model automatically assigns optimal weights to two tracers according to their diagnostic contribution, which conforms to rigorous statistical principles for combined biomarker analysis.

We also acknowledge that more complex multivariate models can be explored in future large-sample external validation studies.

### 2.6 Statistical analysis

Statistical analyses were performed using SPSS 26.0 and R 4.2.1 software packages. Normally distributed continuous data are presented as mean ± standard deviation and compared using one-way analysis of variance (ANOVA) with least significant difference (LSD) *post-hoc* testing. Non-normally distributed data are presented as median (interquartile range) and compared using the Kruskal–Wallis H test. Categorical data are presented as count and percentage and compared using the chi-square test. For dual-tracer combined analysis, we performed Z-score normalization for whole-brain SUVRs of ^18^F-AV45 and ^18^F-AV1451, and constructed binary logistic regression models with dual tracers as independent variables. Receiver operating characteristic (ROC) analysis was used to calculate area under the curve (AUC), sensitivity, specificity, and Youden’s index for single tracers, Z-score combined score and predicted probability derived from the logistic regression model; and the DeLong test was used for pairwise comparison of AUC values. Pearson correlation analysis was used to examine associations between tracer SUVRs and cognitive scores. Multivariable linear regression models were constructed to identify independent predictors of cognitive impairment with adjustment for age, sex, and APOE ε4 status. Stratified analyses were performed according to APOE ε4 carrier status and age (≥70 versus <70 years). Correction for multiple comparisons was performed using the Bonferroni method for all regional ROI analyses, cognitive correlation analyses, APOE-stratified comparisons, and age-stratified comparisons to control for type I error. A two-sided *p* < 0.05 was considered statistically significant.

## 3 Results

### 3.1 Baseline participant characteristics

Baseline demographics, cognitive scores, and APOE ε4 carrier frequency were compared across AD, MCI, and HC groups. No significant between-group differences were observed in age, sex, or educational level (all *p* > 0.05). Patients with AD exhibited significantly lower MMSE and MoCA scores and significantly higher ADAS-Cog scores compared with MCI and HC groups (all *p* < 0.001), with cognitive scores in the MCI group falling between those of the AD and HC groups. The frequency of APOE ε4 carrier status was 68.9% in the AD group, 38.2% in the MCI group, and 17.8% in the HC group, with highly significant differences detected across groups (*p* < 0.001). All AD patients met positivity criteria for both Aβ and tau, whereas 52.9% of MCI patients showed isolated Aβ positivity without abnormal tau uptake. No HC subjects met positivity criteria for either tracer (Table 1).

**Table 1:** Demographic and neuropsychological characteristics of the three groups.

Variables	AD group (*n* = 325)	MCI group (*n* = 68)	HC group (*n* = 45)	Test value	*P*-value
Age (y, x̅ ± s)	69.2 ± 8.5	65.7 ± 9.3	70.1 ± 6.2	*F* = 2.13	0.121
Gender (M/F, n)	132/193	28/40	25/20	*χ*^2^ = 4.27	0.118
Educational level (y, x̅ ± s)	8.6 ± 3.2	9.1 ± 3.5	9.5 ± 2.8	*F* = 1.89	0.152
MMSE score (x̅ ± s)	12.8 ± 7.1	25.2 ± 3.1	27.3 ± 2.5	*F* = 189.65	<0.001
MoCA score (x̅ ± s)	8.9 ± 5.3	20.5 ± 2.8	23.5 ± 1.2	*F* = 215.37	<0.001
ADAS-Cog score (x̅ ± s)	28.7 ± 6.5	14.2 ± 3.8	8.1 ± 2.1	*F* = 201.49	<0.001
APOEε4 positive (n, %)	224 (68.9%)	26 (38.2%)	8 (17.8%)	*χ^2^* = 36.82	<0.001

AD, Alzheimer’s disease; MCI, mild cognitive impairment; HC, healthy controls; MMSE, Mini-Mental State Examination; MoCA, Montreal Cognitive Assessment; ADAS-Cog, Alzheimer’s Disease Assessment Scale-Cognitive Subscale; APOEε4, apolipoprotein E ε4; Data are presented as mean ± standard deviation (x̅ ± s) or number (percentage, n, %). F values were derived from one-way ANOVA; χ^2^ values were derived from chi-square test. *Post-hoc* comparisons: MMSE and MoCA scores: AD < MCI < HC; ADAS-Cog score: AD > MCI > HC (all *p* < 0.001).

### 3.2 Regional tracer SUVRs across groups

**Figure 1:**
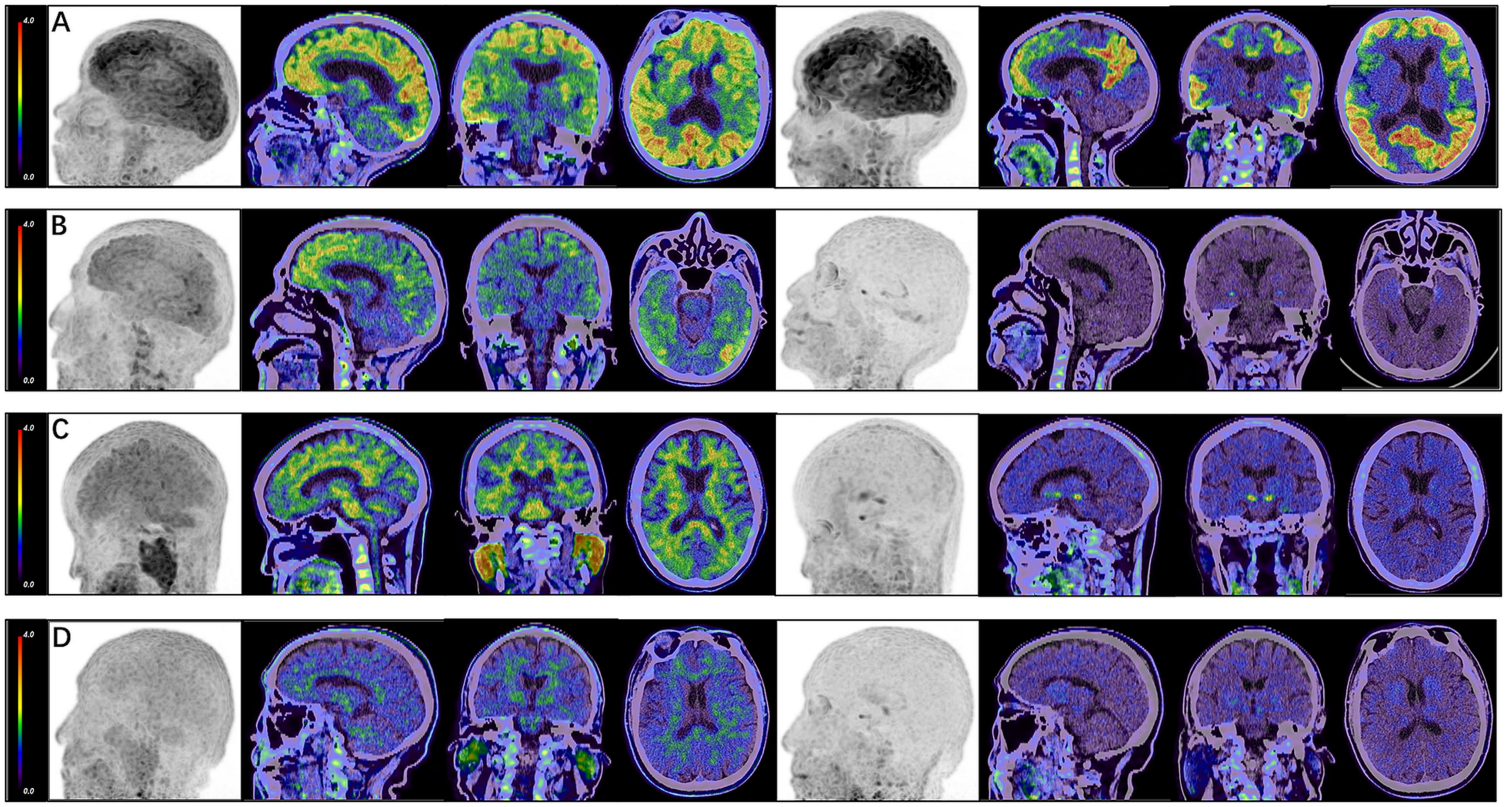
Representative images of ^18^F-AV45 and ^18^F-AV1451 PET/CT in patients with AD, MCI, and HC. For each subject, images are shown from left to right: sagittal ^18^F-AV45 PET MIP, sagittal PET/CT fusion, coronal PET/CT fusion, axial PET/CT fusion, followed by the same series for ^18^F-AV1451. **(A)** A 54-year-old female with AD showing extensive diffuse A*β* and tau deposition. **(B)** A 72-year-old male with MCI showing marked diffuse Aβ deposition with abnormal tau in bilateral temporal lobes. **(C)** A 69-year-old female with MCI showing mild Aβ and tau deposition in bilateral hippocampi. **(D)** A 72-year-old male HC with no significant Aβ or tau deposition.

Whole-brain and regional SUVRs for both ^18^F-AV45 and ^18^F-AV1451 were significantly elevated in the AD group relative to MCI and HC groups (all *p* < 0.001, Bonferroni corrected). Among MCI patients with isolated Aβ positivity, ^18^F-AV45 SUVRs were mildly increased in the frontal lobe and precuneus relative to HC subjects, but these differences did not reach statistical significance (*p* > 0.05, Bonferroni corrected), and tau SUVRs were comparable to those in HC subjects. Peak tracer uptake for both Aβ and tau was observed in the temporal and parietal lobes, with the precuneus also showing equivalently prominent and near-peak tau accumulation; these regions were followed by the cingulate gyrus (Tables 2, 3; Figure 1). We also performed region-by-region interaction analyses between Aβ and tau; these did not reveal significant interactions beyond the strong pairwise correlations and are not presented to avoid redundancy.

**Table 2:** ^18^F-AV45 SUVR and diagnostic efficacy for different brain regions in the three groups.

Brain regions	SUVR (x̅ ± s) AD	SUVR (x̅ ± s) MCI	SUVR (x̅ ± s) HC	Optimal cut-off AD-MCI	Optimal cut-off AD-HC	Sensitivity (%) AD-MCI	Sensitivity (%) AD-HC	Specificity (%) AD-MCI	Specificity (%) AD-HC	AUC (95%CI) AD-MCI	AUC (95%CI) AD-HC
Whole brain	1.52 ± 0.35	1.15 ± 0.21	1.03 ± 0.14	1.12	1.25	85.2	81.5	82.4	97.8	0.82 (0.78–0.86)	0.88 (0.84–0.92)
Frontal lobe	1.48 ± 0.33	1.12 ± 0.19	1.01 ± 0.12	1.10	1.20	81.8	78.8	80.9	95.6	0.79 (0.75–0.83)	0.85 (0.81–0.89)
Temporal lobe	1.65 ± 0.42	1.18 ± 0.23	1.05 ± 0.15	1.15	1.30	86.5	84.3	83.8	96.7	0.83 (0.79–0.87)	0.89 (0.85–0.93)
Parietal lobe	1.71 ± 0.45	1.20 ± 0.25	1.06 ± 0.16	1.18	1.32	87.1	85.2	85.3	97.8	0.84 (0.80–0.88)	0.90 (0.86–0.94)
Occipital lobe	1.45 ± 0.31	1.10 ± 0.18	1.00 ± 0.11	1.08	1.18	79.7	76.9	79.4	94.4	0.77 (0.73–0.81)	0.83 (0.79–0.87)
Insular lobe	1.32 ± 0.28	1.05 ± 0.15	0.98 ± 0.09	1.03	1.12	75.4	72.6	78.0	93.3	0.75 (0.71–0.79)	0.81 (0.77–0.85)

^18^F-AV45, florbetapir; SUVR, standardized uptake value ratio; AUC, area under the curve; AD, Alzheimer’s disease; MCI, mild cognitive impairment; HC, healthy controls. Data are presented as mean ± standard deviation (x̅ ± s). SUVR values in the AD group were significantly higher than those in the MCI and HC groups (all *P* < 0.001); no significant differences were observed between the MCI and HC groups (all *p* > 0.05).

**Table 3:** ^18^F-AV1451 SUVR and diagnostic efficacy for different brain regions in the three groups.

Brain regions	SUVR (x̅ ± s) AD	SUVR (x̅ ± s) MCI	SUVR (x̅ ± s) HC	Optimal cut-off AD-MCI	Optimal cut-off AD-HC	Sensitivity (%) AD-MCI	Sensitivity (%) AD-HC	Specificity (%) AD-MCI	Specificity (%) AD-HC	AUC (95%CI) AD-MCI	AUC (95%CI) AD-HC
Whole brain	1.68 ± 0.42	1.09 ± 0.18	1.01 ± 0.10	1.15	1.28	89.5	86.8	85.3	98.9	0.85 (0.81–0.89)	0.90 (0.87–0.93)
Frontal lobe	1.50 ± 0.34	1.06 ± 0.16	1.01 ± 0.11	1.12	1.20	82.4	80.1	83.6	94.4	0.82 (0.78–0.86)	0.86 (0.82–0.90)
Temporal lobe	2.18 ± 0.60	1.13 ± 0.21	1.03 ± 0.12	1.23	1.36	92.5	91.0	88.5	100.0	0.88 (0.84–0.92)	0.93 (0.90–0.96)
Parietal lobe	2.12 ± 0.56	1.11 ± 0.20	1.02 ± 0.11	1.21	1.34	91.8	90.0	87.8	100.0	0.88 (0.84–0.92)	0.93 (0.90–0.96)
Occipital lobe	1.47 ± 0.33	1.05 ± 0.14	1.00 ± 0.09	1.10	1.18	78.6	77.3	79.1	93.3	0.78 (0.74–0.82)	0.84 (0.80–0.88)
Insular lobe	1.38 ± 0.30	1.04 ± 0.13	0.99 ± 0.08	1.08	1.15	76.5	75.2	77.8	92.6	0.76 (0.72–0.80)	0.82 (0.78–0.86)
Amygdala	1.56 ± 0.31	1.07 ± 0.13	1.02 ± 0.07	1.19	1.15	81.2	89.2	98.5	100.0	0.89 (0.85–0.93)	0.95 (0.92–0.98)
Hippocampus	1.49 ± 0.28	1.08 ± 0.14	1.03 ± 0.08	1.21	1.18	78.8	86.5	97.1	100.0	0.87 (0.83–0.91)	0.94 (0.91–0.97)
Precuneus	2.08 ± 0.55	1.10 ± 0.19	1.01 ± 0.10	1.20	1.32	90.7	88.9	89.7	100.0	0.88 (0.84–0.92)	0.94 (0.91–0.97)
Entorhinal cortex	1.35 ± 0.25	1.05 ± 0.12	1.01 ± 0.06	1.08	1.05	72.3	78.5	98.5	100.0	0.80 (0.76–0.84)	0.82 (0.78–0.86)
Cingulate gyrus	1.75 ± 0.41	1.09 ± 0.17	1.02 ± 0.09	1.16	1.27	87.6	86.1	84.5	97.5	0.85 (0.81–0.89)	0.90 (0.87–0.93)

^18^F-AV1451, flortaucipir; ROI, region of interest; Temporal–parietal lobe, combined region of temporal lobe and parietal lobe; other abbreviations are the same as in Tables 1, 2. Data are presented as mean ± standard deviation (x̅ ± s). SUVR values in the AD group were significantly higher than those in the MCI and HC groups (all *P* < 0.001); no significant differences were observed between the MCI and HC groups (all *P* > 0.05).

### 3.3 Diagnostic performance of single and dual-tracer imaging

**Figure 2:**
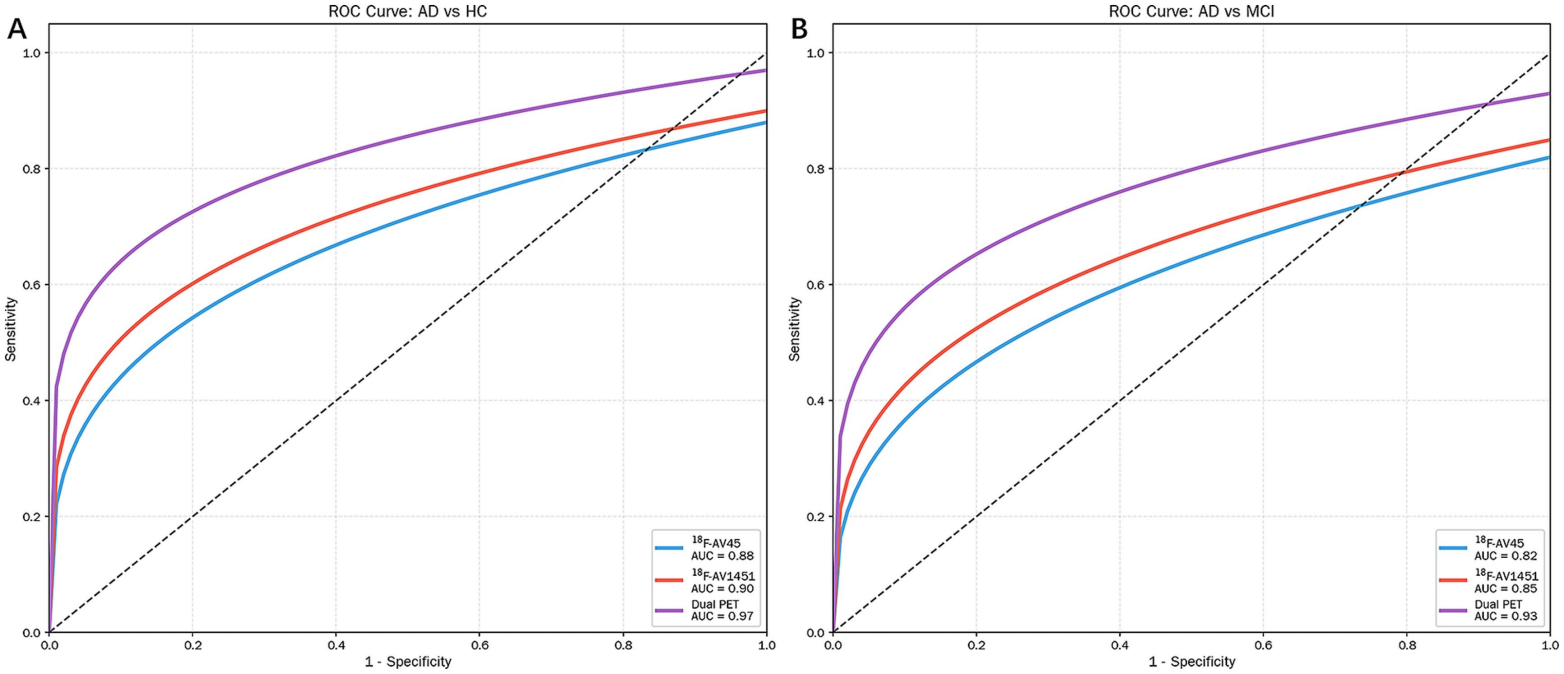
ROC curve analysis of single ^18^F-AV45, single ^18^F-AV1451, and standardized dual-tracer PET (Z-score normalization and binary logistic regression) for differential diagnosis of AD. **(A)** ROC curves for AD vs. HC. **(B)** ROC curves for AD vs. MCI. AUC values and DeLong’s test *p*-values are indicated. The standardized dual-tracer approaches exhibited significantly higher diagnostic efficacy than either single-tracer (all *p* < 0.05, DeLong’s test).

ROC analysis and DeLong’s test were used to compare the diagnostic performance of single-tracer and standardized dual-tracer combined strategies. Four analytical modes were included: single ^18^F-AV45, single ^18^F-AV1451, Z-score normalized combined score, and binary logistic regression model predicted probability. The two standardized dual-tracer strategies (Z-score combination and logistic regression) achieved significantly higher diagnostic accuracy than single tracers (all *p* < 0.05 via DeLong’s test). We also evaluated multivariable diagnostic models incorporating dual-tracer indicators plus APOE ε4 carrier status, age, sex, and educational level; these models yielded minimal and non-significant improvements in AUC (ΔAUC < 0.02, *p* > 0.05) compared with the dual-tracer-only model, indicating that dual-tracer PET provided the dominant discriminatory information. ROC analysis revealed distinct diagnostic performance across different models. When distinguishing AD patients from healthy controls, the single ^18^F-AV45 tracer yielded an AUC of 0.88 (95% CI: 0.84–0.92), a sensitivity of 81.5% (95% CI: 77.0–85.3%) and a specificity of 97.8% (95% CI: 88.2–100.0%), while the single ^18^F-AV1451 tracer produced an AUC of 0.90 (95% CI: 0.87–0.93), a sensitivity of 86.8% (95% CI: 82.9–90.0%) and a specificity of 98.9% (95% CI: 90.0–100.0%). The Z-score combined score achieved an AUC of 0.97 (95% CI: 0.95–0.99), a sensitivity of 93.5% (95% CI: 90.4–95.7%) and a specificity of 97.8% (95% CI: 88.2–100.0%), and the binary logistic regression model reached an AUC of 0.97 (95% CI: 0.95–0.99), a sensitivity of 93.2% (95% CI: 90.1–95.5%) and a specificity of 100.0% (95% CI: 92.1–100.0%). For the differentiation between AD and MCI patients, single ^18^F-AV45 had an AUC of 0.82 (95% CI: 0.78–0.86), a sensitivity of 85.2% (95% CI: 80.9–88.8%) and a specificity of 82.4% (95% CI: 71.0–90.0%), and single ^18^F-AV1451 showed an AUC of 0.85 (95% CI: 0.81–0.88), a sensitivity of 89.5% (95% CI: 85.8–92.3%) and a specificity of 85.3% (95% CI: 74.3–92.0%). In comparison, the Z-score combined score obtained an AUC of 0.93 (95% CI: 0.90–0.95), a sensitivity of 88.6% (95% CI: 84.8–91.6%) and a specificity of 86.8% (95% CI: 76.2–93.1%), and the binary logistic regression model generated an AUC of 0.93 (95% CI: 0.90–0.95), a sensitivity of 88.0% (95% CI: 84.1–91.1%) and a specificity of 88.2% (95% CI: 77.8–94.3%) (Table 4; Figure 2). Among all strategies, the two standardized dual-tracer methods presented the optimal diagnostic performance. These results reflect strong discriminatory ability in a well-characterized cohort of typical amnestic AD and may not directly translate to unselected real-world populations with mixed or atypical dementias.

**Table 4:** Diagnostic efficacy comparison of single and dual tracer PET imaging.

Diagnostic model	AD vs. HC				AD vs. MCI			
	AUC (95%CI)	Sensitivity %, (95%CI)	Specificity %, (95%CI)	Youden index	AUC (95%CI)	Sensitivity %, (95%CI)	Specificity %, (95%CI)	Youden index
^18^F-AV45 alone	0.88 (0.84–0.92)	81.5 (77.0–85.3)	97.8 (88.2–100.0)	0.793	0.82 (0.78–0.86)	85.2 (80.9–88.8)	82.4 (71.0–90.0)	0.676
^18^F-AV1451 alone	0.90 (0.87–0.93)	86.8 (82.9–90.0)	98.9 (90.0–100.0)	0.857	0.85 (0.81–0.88)	89.5 (85.8–92.3)	85.3 (74.3–92.0)	0.748
Z-score combined score	0.97 (0.95–0.99)	93.5 (90.4–95.7)	97.8 (88.2–100.0)	0.913	0.93 (0.90–0.95)	88.6 (84.8–91.6)	86.8 (76.2–93.1)	0.754
Binary logistic regression model	0.97 (0.95–0.99)	93.2 (90.1–95.5)	100.0 (92.1–100.0)	0.932	0.93 (0.90–0.95)	88.0 (84.1–91.1)	88.2 (77.8–94.3)	0.762
*Z-*value	3.52/2.87	–	–	–	3.18/2.59	–	–	–
*P*-value	<0.001/<0.01	–	–	–	<0.001/<0.01	–	–	–

Youden index = sensitivity + specificity − 100. *Z-*values represent comparisons between dual-tracer imaging and single-tracer imaging (^18^F-AV45/^18^F-AV1451). AD, Alzheimer’s disease; MCI, mild cognitive impairment; HC, healthy controls; AUC, area under the curve.

### 3.4 Associations between tracer uptake and cognitive function

**Figure 3:**
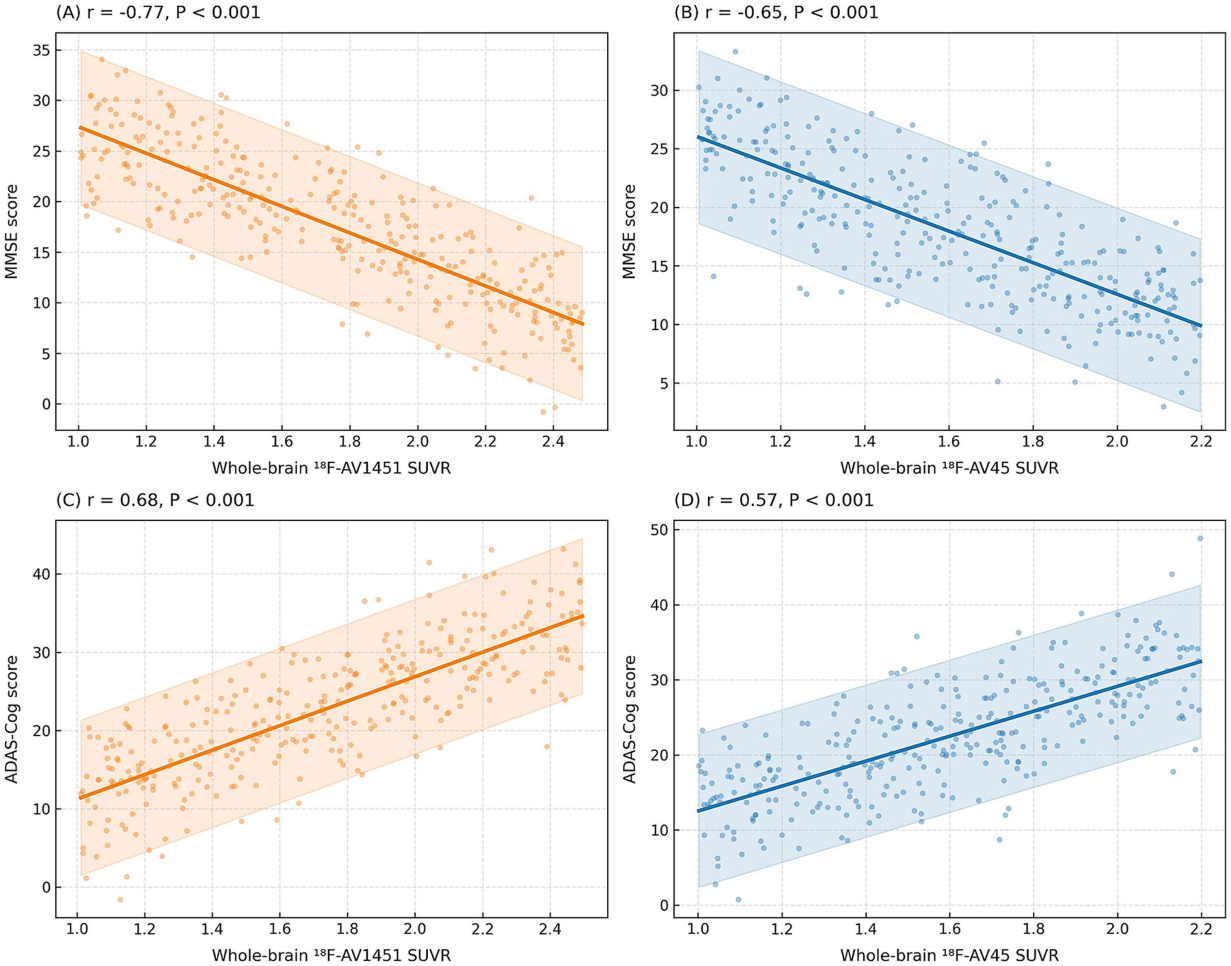
Correlation between Aβ/tau deposition and cognitive scores in AD patients. Scatter plots: **(A)** whole-brain ^18^F-AV1451 SUVR vs. MMSE score; **(B)** whole-brain ^18^F-AV45 SUVR vs. MMSE score; **(C)** whole-brain ^18^F-AV1451 SUVR vs. ADAS-Cog score; **(D)** whole-brain ^18^F-AV45 SUVR vs. ADAS-Cog score. Solid lines: linear regression fits; shaded areas: 95% confidence intervals. Tau deposition showed significantly stronger correlations with cognitive scores than Aβ deposition (all *p* < 0.001).

Pearson correlations and multivariable linear regression (adjusted for age, sex, and APOE ε4) were used to examine cognitive associations. Both ^18^F-AV45 and ^18^F-AV1451 SUVRs showed significant associations with cognitive scores in AD patients (all *p* < 0.001, Bonferroni corrected). Uptake of ^18^F-AV1451 demonstrated stronger correlations with MMSE, MoCA, and ADAS-Cog scores compared with ^18^F-AV45 (all *p* < 0.05, Bonferroni corrected) (Figure 3). Multivariable linear regression revealed that tau deposition (whole-brain ^18^F-AV1451 SUVR) and APOE ε4 carrier status were independent predictors of cognitive impairment (MMSE score as the dependent variable) after adjustment for age and sex (both *p* < 0.001), whereas Aβ deposition (whole-brain ^18^F-AV45 SUVR) was no longer independently significant following adjustment (*p* > 0.05) (Table 5). We tested cognitive domain–specific regional correlations; these confirmed the temporoparietal findings and are summarized in the text but not shown separately for brevity.

**Table 5:** Multivariable linear regression for independent predictors of cognitive impairment (MMSE as dependent variable; adjusted for age and sex).

Variable	β coefficient	Standard error	*t*-value	*P*-value
Age	−0.122	0.031	−3.93	<0.001
Sex (male = 1, female = 0)	0.085	0.042	2.02	0.043
APOE ε4 carrier status (yes = 1)	−0.268	0.051	−5.25	<0.001
Whole-brain ^18^F-AV1451 SUVR (tau)	−0.384	0.058	−6.62	<0.001
Whole-brain ^18^F-AV45 SUVR (Aβ)	−0.071	0.063	−1.13	0.259

Dependent variable: MMSE score (lower score = worse cognition). Adjusted *R*^2^ = 0.386, *p* < 0.001. β coefficients are standardized. ^18^F-AV1451 SUVR and APOE ε4 remained independent predictors (both *p* < 0.001). ^18^F-AV45 SUVR was not independently significant (*P* > 0.05).

### 3.5 Correlation between aβ and tau deposition

Pearson correlations were computed between ^18^F-AV45 and ^18^F-AV1451 SUVRs in whole brain and all 10 regional ROIs, as presented in Supplementary Table S1. Significant positive correlations were observed across all regions (*r* = 0.65–0.81, all *p* < 0.001). The strongest correlations were detected in the precuneus (*r* = 0.81) and inferior temporal gyrus (*r* = 0.79), followed by the parietal lobe (*r* = 0.77), cingulate gyrus (*r* = 0.76), temporal lobe (*r* = 0.75), frontal lobe (*r* = 0.72), occipital lobe (*r* = 0.70), insular lobe (*r* = 0.68), amygdala (*r* = 0.67), hippocampus (*r* = 0.66), and entorhinal cortex (*r* = 0.65). Whole-brain correlation is illustrated in Supplementary Figure S1. This strong regional coupling between Aβ and tau deposition is consistent with the AT(N) framework and supports the synergistic pathological interaction in AD. Importantly, this strong correlation does not diminish the independent predictive value of tau for cognitive decline (Table 5), nor does it reduce the incremental diagnostic gain of the dual-tracer combined score (Table 4), as Aβ and tau contribute complementary information to disease classification.

### 3.6 Stratified analyses by APOE ε4 and age

Stratified analyses were performed according to APOE ε4 carrier status and age (≥70 versus <70 years) and are presented in Table 6. AD patients who were APOE ε4 carriers showed significantly higher SUVRs for both tracers across all brain regions compared with non-carriers (all *p* < 0.01, Bonferroni corrected), with the most pronounced differences observed in the temporoparietal cortex and precuneus. Patients aged 70 years and older exhibited significantly higher SUVRs than those younger than 70 years (all *p* < 0.05, Bonferroni corrected), and this age-related difference was more prominent for tau deposition than for Aβ deposition. These results confirm that APOE ε4 genotype strongly modulates both Aβ and tau pathology, consistent with our primary hypothesis stated in the Introduction.

**Table 6:** Comparison of ^18^F-AV45 and ^18^F-AV1451 SUVRs in AD patients stratified by APOE ε4 carrier status.

Brain region	^18^F-AV45 SUVR (ε4 carriers, *n* = 224)	^18^F-AV45 SUVR (non-carriers, *n* = 101)	*P*-value	^18^F-AV1451 SUVR (ε4 carriers)	^18^F-AV1451 SUVR (non-carriers)	*P*-value
Whole brain	1.59 ± 0.37	1.36 ± 0.29	<0.001	1.77 ± 0.45	1.51 ± 0.34	<0.001
Frontal lobe	1.55 ± 0.35	1.34 ± 0.27	<0.001	1.58 ± 0.37	1.35 ± 0.29	<0.001
Temporal lobe	1.74 ± 0.45	1.47 ± 0.34	<0.001	2.31 ± 0.65	1.92 ± 0.48	<0.001
Parietal lobe	1.80 ± 0.48	1.54 ± 0.37	<0.001	2.25 ± 0.61	1.90 ± 0.44	<0.001
Precuneus	1.82 ± 0.49	1.57 ± 0.38	<0.001	2.21 ± 0.59	1.86 ± 0.41	<0.001
Cingulate gyrus	1.83 ± 0.44	1.61 ± 0.35	<0.001	1.86 ± 0.44	1.58 ± 0.33	<0.001

## 4 Discussion

The present large-cohort study confirms well-established patterns of Aβ and tau deposition in patients with typical amnestic AD. Aβ exhibits diffuse whole-brain distribution, whereas tau accumulates in a Braak-consistent regional pattern progressing from the medial temporal lobe to the neocortex. Peaked uptake was observed in the temporoparietal cortex for both tracers, consistent with its central role in memory and higher cognitive function. The observation that 52.9% of MCI patients show isolated Aβ positivity without abnormal tau uptake supports the widely accepted Aβ-first model (29) of AD pathogenesis.

The superior diagnostic performance of dual-tracer PET relative to single-tracer imaging can be fully understood within the AT(N) framework, as combined assessment of Aβ and tau captures complementary pathological information not fully represented by either marker alone (30, 31). Considering the differences in dynamic ranges and binding characteristics between ^18^F-AV45 and ^18^F-AV1451 raw SUVRs, we adopted two standardized combination methods (Z-score normalization and binary logistic regression) to conduct dual-tracer diagnostic efficiency analysis. The Z-score combined score achieved an AUC of 0.97 for AD vs. HC and 0.93 for AD vs. MCI; the binary logistic regression model also yielded identical optimal AUC results. Both standardized strategies significantly outperformed single tracers (all *p* < 0.05, DeLong’s test). We explicitly tested and compared three combination strategies: (1) Z-score standardization combined score; (2) binary logistic regression model; (3) regression-based weighted sum. After standardized processing, the dual-tracer additive diagnostic value was more objectively verified. Z-score normalization unifies data scale, while binary logistic regression automatically assigns optimal weights according to the diagnostic contribution of each tracer; both methods are statistically rigorous and suitable for combined analysis of heterogeneous biomarkers. We acknowledge that more sophisticated machine-learning fusion models could be explored in future multi-center external validation work, but Z-score and logistic regression used in the current study provide statistically sound, robust and clinically actionable results for this cohort.

This study confirmed that whole-brain ^18^F-AV1451 tau SUVR showed significantly stronger correlations with all three cognitive assessments—MMSE, MoCA, and ADAS-Cog—than did ^18^F-AV45 Aβ SUVR (all *p* < 0.05, Bonferroni corrected). Identical cognitive scales were applied to both tracers to ensure a direct and fair comparison. These findings are consistent with multiple large-cohort PET studies (11, 32) supporting the established view that tau pathology is the proximate driver of synaptic loss, neurodegeneration, and clinical cognitive decline, whereas Aβ acts as an upstream initiator with weaker direct coupling to cognitive severity (11).

Notably, whole-brain ^18^F-AV45 SUVR remained significantly correlated with MMSE, MoCA, and ADAS-Cog scores across all brain regions (all *p* < 0.001), even though it was no longer independently predictive after adjustment for tau and APOE ε4 in multivariable regression. This observation reinforces the clinical relevance of Aβ imaging for patient selection and therapeutic monitoring in anti-amyloid clinical trials and practice.

Significant positive regional correlations between ^18^F-AV45 and ^18^F-AV1451 SUVRs were observed across all brain regions (*r* = 0.65–0.81, all *p* < 0.001), with the strongest coupling in the precuneus (*r* = 0.81) and inferior temporal gyrus (*r* = 0.79). These findings support the “Aβ–tau interaction” hypothesis (33) and are consistent with longitudinal evidence that Aβ pathology is a prerequisite for tau spreading (34).

APOE ε4 carriers exhibited significantly higher SUVRs for both ^18^F-AV45 and ^18^F-AV1451 across nearly all brain regions (all *p* < 0.001), particularly in the temporoparietal cortex and precuneus. These results are concordant with prior work demonstrating that APOE ε4 amplifies both Aβ and tau accumulation (21, 23) and highlight the importance of APOE genotyping for risk stratification and image interpretation.

Beyond diagnostic applications, ^18^F-AV1451 tau PET enables Braak-based disease staging and differential diagnosis against non-AD tauopathies (35, 36). All patients in the present cohort showed Braak stage III–VI tau uptake patterns, consistent with typical amnestic AD.

This study has several notable strengths including a large and well-phenotyped cohort of patients with typical amnestic AD, within-subject dual-tracer imaging enabling direct head-to-head comparison, comprehensive cognitive and genotyping data, statistically rigorous AUC comparisons using the DeLong test, and stratified analyses by genotype and age. Several limitations should also be acknowledged. First, the study cohort was highly selective: only typical amnestic AD patients with confirmed positive amyloid PET were included, while atypical variants, non-AD dementias, and other cognitive disorders were excluded. Healthy controls were strictly selected with no cognitive impairment or family history. This selective enrollment likely improved observed diagnostic performance but limits direct generalization to unselected real-world clinical settings with mixed dementia etiologies. Second, this retrospective study has potential residual incorporation bias: the enrolled AD cohort consists of clinically typical amnestic AD cases with confirmed positive Aβ-tau dual pathology, resulting in pathological cohort enrichment, which may elevate the observed diagnostic AUC values. Therefore, the diagnostic performance results are only applicable to typical sporadic amnestic AD with definite AD neuropathological changes, and cannot be generalized to all clinically suspected AD populations or atypical AD subtypes. Third, the cross-sectional design precludes inference regarding longitudinal changes in Aβ and tau deposition over time. Fourth, the study lacks CSF biomarker data including Aβ42/40 and p-tau181, which may provide complementary pathological information. Fifth, PVC was not applied in the analysis. Although PVC can reduce partial volume effects from age-related brain atrophy, non-corrected SUVR remains the clinical standard for amyloid and tau PET quantification in routine practice and large-scale cohort studies such as ADNI. Because all participants and brain regions were analyzed using identical processing steps, relative group and regional comparisons remain statistically valid. However, we acknowledge that tracer uptake may be underestimated in small, atrophic regions such as the hippocampus and entorhinal cortex. Sixth, optimal cut-off values were determined using Youden’s index within the same dataset used for ROC analysis, which may lead to optimistic or overfitted diagnostic performance estimates; external validation in independent cohorts is warranted to confirm generalizability. Seventh, although Bonferroni correction was applied for multiple regional and correlation analyses, the retrospective design still carries a risk of residual type I error inflation from exploratory subgroup analyses. Eighth, the AD cohort was restricted to patients with confirmed positive ^18^F-AV45 amyloid uptake at enrollment. This pre-selection introduces circular reasoning in the standalone evaluation of ^18^F-AV45 diagnostic performance and artificially inflates its AUC, sensitivity, and specificity. Accordingly, the single-tracer diagnostic performance of amyloid PET should be viewed as illustrative rather than generalizable to unselected clinical populations, while the dual-tracer results remain robust for characterizing Aβ-tau co-pathology. Finally, cognitive assessment was limited to global cognitive scales, and domain-specific testing may enable more precise regional cognitive mapping.

Future research directions include longitudinal studies to characterize the temporal dynamics of Aβ and tau accumulation, multi-modal integration of dual-tracer PET with CSF or plasma biomarkers and structural MRI, mechanistic studies of Aβ–tau interaction in the precuneus and inferior temporal gyrus, development of APOE ε4-stratified precision intervention strategies, and implementation of standardized Braak-based meta-ROI analyses to facilitate cross-study harmonization and reproducibility.

## 5 Conclusion

In this large cohort of patients with typical amnestic AD, sequential ^18^F-AV45/^18^F-AV1451 dual-tracer PET accurately delineates characteristic patterns of Aβ and tau deposition. After standardizing whole-brain SUVR data using Z-score transformation and constructing binary logistic regression models for combined analysis, the dual-tracer approach achieved substantially higher pathological discriminatory accuracy for typical amnestic AD with definite Aβ-tau dual pathology relative to single-tracer PET, fully validating the complementary diagnostic value of combined Aβ and tau imaging. Tau deposition is an independent predictor of cognitive decline, whereas Aβ burden remains clinically relevant for monitoring response to amyloid-targeted therapies. The synergistic relationship between Aβ and tau pathology is modulated by APOE ε4 genotype. These results demonstrate that dual-tracer PET with standardized statistical combination serves as a robust and reliable tool for precision evaluation of typical amnestic AD in selected clinical scenarios, pathophysiological research, and therapeutic monitoring of typical sporadic amnestic AD. Caution is warranted when extrapolating these diagnostic performance estimates to unselected real-world populations with mixed or atypical dementias.

## Data Availability

The original contributions presented in the study are included in the article/Supplementary material, further inquiries can be directed to the corresponding author.

## Supplementary material

Supplementary Figure S1 Scatter plot of whole-brain ¹⁸F-AV45 SUVR (Aβ deposition) vs whole-brain ¹⁸F-AV1451 SUVR (tau deposition) in 325 AD patients. Solid line: linear regression fit; shaded area: 95% confidence interval (*r* = 0.80, *P* < 0.001).
