# Supplementary figures and images for: Sequential 18F-AV45/18F-AV1451 dual-tracer brain PET imaging in Alzheimer’s disease: amyloid-tau deposition, diagnostic performance, cognitive associations, and modulation by APOE ε4
Source: Front Neurol. 2026 Jun 19;17:1877217. doi: 10.3389/fneur.2026.1877217 (PMC13327867; doi:10.3389/fneur.2026.1877217)

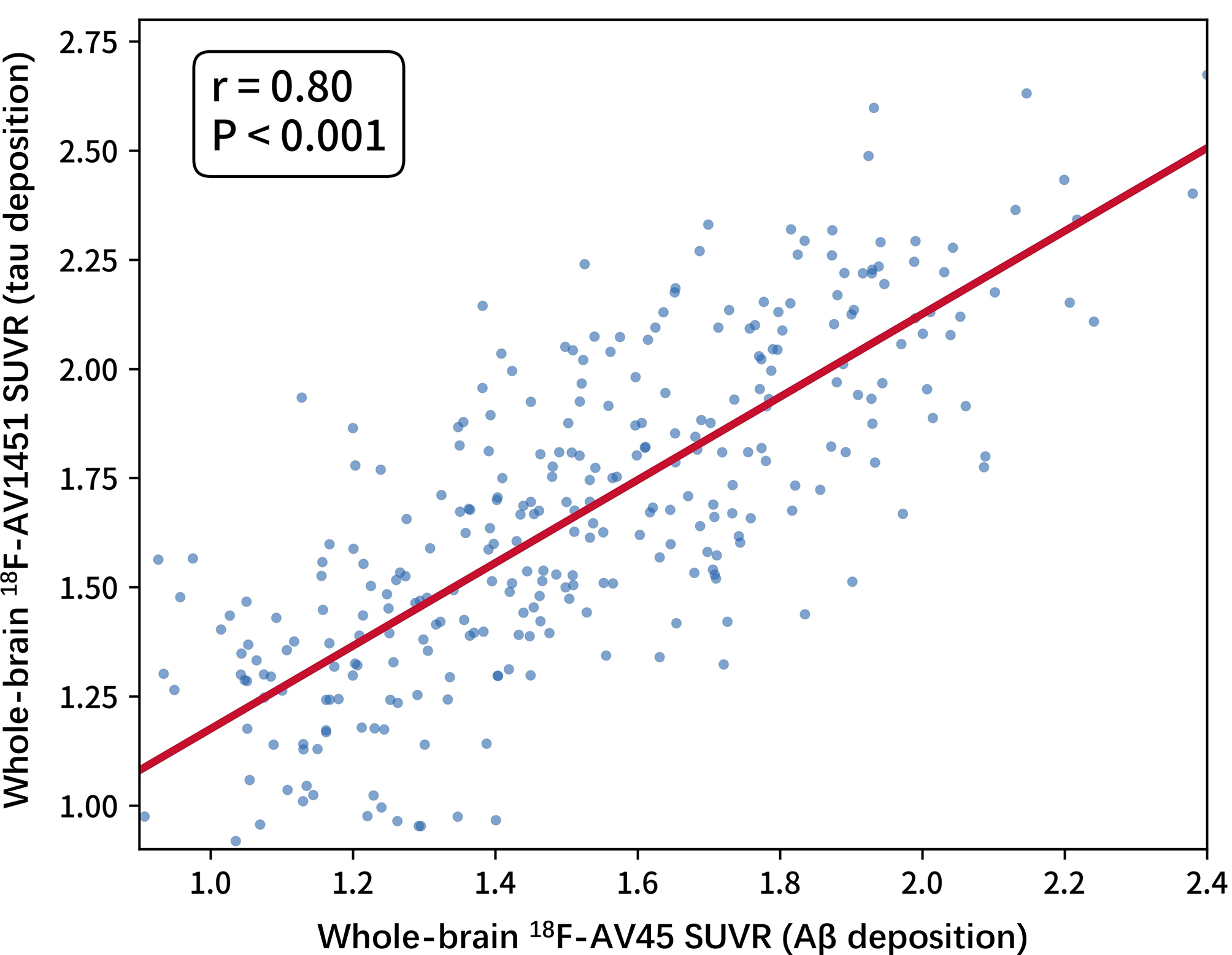

Supplement: Supplementary Figure S1 — Scatter plot of whole-brain ¹⁸F-AV45 SUVR (Aβ deposition) vs whole-brain ¹⁸F-AV1451 SUVR (tau deposition) in 325 AD patients. Solid line: linear regression fit; shaded area: 95% confidence interval (r = 0.80, P < 0.001). [file Image_1.tif]
