# Supplementary material for: Sequential 18F-AV45/18F-AV1451 dual-tracer brain PET imaging in Alzheimer’s disease: amyloid-tau deposition, diagnostic performance, cognitive associations, and modulation by APOE ε4
Source: Front Neurol. 2026 Jun 19;17:1877217. doi: 10.3389/fneur.2026.1877217 (PMC13327867; doi:10.3389/fneur.2026.1877217)
Supplement: Supplementary file 2 [file Table_1.docx]

**TABLE S1. Regional Pearson correlation coefficients (*r*) between ¹⁸F‑AV45 SUVR (Aβ) and ¹⁸F‑AV1451 SUVR (tau).**

| Brain Region | *r* value | *P* value |
| --- | --- | --- |
| Precuneus | 0.81 | < 0.001 |
| Inferior temporal gyrus | 0.79 | < 0.001 |
| Parietal lobe | 0.77 | < 0.001 |
| Cingulate gyrus | 0.76 | < 0.001 |
| Temporal lobe | 0.75 | < 0.001 |
| Frontal lobe | 0.72 | < 0.001 |
| Occipital lobe | 0.70 | < 0.001 |
| Insular lobe | 0.68 | < 0.001 |
| Amygdala | 0.67 | < 0.001 |
| Hippocampus | 0.66 | < 0.001 |
| Entorhinal cortex | 0.65 | < 0.001 |
| Whole brain | 0.80 | < 0.001 |
